# Effect of telephone counselling on the knowledge, attitude and practices of contacts of confirmed COVID-19 cases in Egypt

**DOI:** 10.4102/phcfm.v13i1.2852

**Published:** 2021-07-12

**Authors:** Randa M. Said, Ghada M. Salem

**Affiliations:** 1 Department of Family Medicine, Faculty of Medicine, Zagazig University, Zagazig, Egypt; 2 Department of Public Health and Community Medicine, Faculty of Medicine, Zagazig University, Zagazig, Egypt

**Keywords:** telephone counselling, KAP score, contacts of confirmed COVID-19-cases, COVID-19 stigma, routine surveillance, health education, home isolation

## Abstract

**Background:**

The coronavirus disease 2019 (COVID-19) is an emerging respiratory illness. The World Health Organization declared it a public health emergency of international concern on 30 January 2020 and called for collaborative efforts, such as contact tracing and promoting the public awareness about COVID-19, and recommended prevention and control measures.

**Aim:**

The aim of this study was to assess the effect of telephone counselling on the knowledge, attitude and practices (KAPs) of contacts of COVID-19 confirmed cases towards COVID-19 epidemiology and infection prevention and control measures.

**Setting:**

Ten areas in Sharkia Governorate, Egypt divided into six rural and four urban areas.

**Methods:**

A non-randomised controlled trial was conducted in Sharkia Governorate, Egypt, from 26 March 2020 to 12 April 2020 on 208 contacts of confirmed COVID-19 cases, divided equally into two groups: an experiment group that was exposed to telephone counselling by the researchers and a control group that was exposed to routine surveillance by local health authority. A semi-structured questionnaire was used to assess the KAP of both groups towards COVID-19 before and after intervention.

**Results:**

After intervention the percent of contacts who achieved good knowledge, positive attitudes and better practice scores in the experimental group was 91.3%, 57.8% and 71.2%, respectively, compared with 13.5%, 7.8% and 16.3%, respectively, in the control group. Male gender and working group were significantly associated with bad practice score. Furthermore, there was a statistically significant positive correlation between differences in knowledge, attitudes and practices of the experimental group before and after the intervention.

**Conclusion:**

This study proved the effectiveness of telephone counselling in improving COVID-19-related KAP scores of contacts of confirmed COVID-19 cases.

## Introduction

The coronavirus disease 2019 (COVID-19) is an emerging respiratory illness that was first identified in December 2019 in Wuhan, China. The disease is highly infectious, leading to a worldwide pandemic. The World Health Organization (WHO) declared it a public health emergency of international concern on 30 January 2020 and called for collaborative efforts of all countries to prevent the rapid spread of COVID-19.^1^

These collaborative efforts include contact tracing and promoting the public awareness about the updates of COVID-19 situation and the recommended prevention and control measures.^2^ Contact tracing has proven to be effective in controlling sporadic and clusters of cases before they can spread infection.^3^ Adherence of the public in general and contacts of COVID-19 confirmed cases in particular to prevention and control measures is the mainstay in the battle against COVID-19; therefore, it is necessary to promote this adherence through health education programmes. Numerous studies have shown that health education can change knowledge, unhealthy attitudes and behaviours and can effectively reduce infectious diseases and epidemics.^4^ Health education will largely affect the knowledge, attitude and practices (KAPs) of target audiences towards COVID-19 according to the KAP theory.^5,6^

According to the WHO, application of electronic means in providing health services is known as E-health. It is one of the ways to provide health care to people who have difficulty to reach health services because of physical or environmental barriers, insufficient resources or when face-to-face health education measures are not feasible during infection outbreaks.^7^ There are many varieties of E-health ranging from simple techniques (telephone and facsimile machines) to complex techniques (satellite links and integrated services digital network). Telephone calls are considered to be one of the simplest and least expensive methods to provide health information, education and psychological support for members of the public.^8^ The effect of telephone counselling is varied; some studies have supported its positive effect, such as the one carried out by Zhu et al.,^9^ who proved the effectiveness of telephone counselling in smoking cessation, whilst other studies such as that of Allagher et al. did not find any evidence to support the positive effects of telephone counselling.^10^

At this critical moment, there is an urgent need to prevent the spread of the COVID-19 infection through increasing awareness of contacts of COVID-19 cases towards infection prevention and control measures. Our research question was the following:

- Can telephone counselling as an easy, inexpensive, and safe method for both health care providers and exposed people increase the abilities of COVID-19 contacts to make informed decisions towards infection prevention and control measures, without succumbing to mistrust and the stigma associated with the disease?

Our study objective was to assess the effect of telephone counselling on KAP of contacts of COVID-19 confirmed cases towards COVID-19 epidemiology and infection prevention and control measures.

## Research methods and design

### Study design

A non-randomised controlled trial was conducted in all areas with confirmed cases of COVID-19 in Sharkia Governorate from 26 March 2020 to 12 April 2020. They included 10 areas divided into six rural and four urban areas.

### Study subjects

The study subjects were contacts of confirmed cases of COVID-19 traced by the local health authorities (LHAs) during the study period. Local health authorities defined a contact as a person who has experienced any one of the following exposures during the two days before and the 14 days after the onset of symptoms of a confirmed case: (1) face-to-face contact with a confirmed case within 1 m and for more than 15 min, (2) direct physical contact with a confirmed case and (3) direct care for a patient with confirmed COVID-19 without using proper personal protective equipment.^11^

### Exclusion criteria

The coronavirus disease 2019 contacts excluded from the study participants less than 18 years old and those living outside the Sharkia Governorate.

### Sample size and sampling method

Sample size was calculated to be 182 participants using OpenEpi program depending on the following: confidence interval 95%, power of test 80%, ratio between two groups 1:1, percent of participants with improved KAP scores in the control group compared with intervention group was 60% and 80%, respectively, according to the pilot study, odds ratio 2.7 and risk ratio 1.3. Taking into consideration a dropout of 20%, the total calculated sample size was 218 participants divided equally between two study groups.

All COVID-19 contacts appearing during the study period were included in the study. There were 208 participants who were divided equally into two groups: an experimental group and a control group. They did not appear at the same time but appeared in consecutive clusters depending on appearance of confirmed COVID-19 cases at different times and in different places. So, the first six clusters were chosen to represent the control group, whilst the next four clusters were chosen to represent the experimental group. The number of clusters depended on trying to approximate the calculated sample size of each group.

### Tools of data collection

#### COVID-19 knowledge, attitude and practices questionnaire

It was a semi-structured questionnaire adapted from Zhong et al.^12^ It was modified by addition of new questions in the attitudes and practices sections so as to be suitable for contacts of COVID-19 cases. It consisted of four parts (items of parts II, III, IV are illustrated in Appendix 1, Table 1-A1).

**Part I:** Socio-demographic characteristics including age, sex, residence, marital state, education, occupation and social class which were calculated according to Fahmy Socioeconomic Level Questionnaire.^13^

**Part II:** There were 12 knowledge questions: four regarding clinical presentations (K1–K4), three regarding transmission routes (K5–K7) and five regarding prevention and control of COVID-19 (K8–K12). These questions were answered on a true/false basis, with an additional ‘I don’t know’ option. A correct answer was given score ‘1’ indicating good knowledge and incorrect answer or ‘I don’t know’ option was given score ‘0’, indicating bad knowledge. The total knowledge score ranged from 0 to 12, with consideration of achieving more than 60% from total score as a good knowledge of COVID-19.

**Part III:** Four attitude questions (A1–A4) assessed the degree of worry amongst COVID-19 contacts about the following: getting the disease, people talking badly about them, avoiding them by friends and family because of being a contact of COVID-19 case and seeking medical care if they have any symptoms suspicious of COVID-19 because of fear of stigma. These questions were answered on a ‘yes/no’ basis, where ‘Yes’ indicated a negative attitude and was scored ‘0’ and ‘No’ indicated a positive attitude and was scored ‘1’. The total attitude score ranged from 0 to 4, with consideration of achieving more than 50% from total score as positive attitude.

**Part IV:** Ten practice questions (P1–P10) assessed the recommended health practices that should be followed by contacts of COVID-19 cases. These questions were answered on a ‘yes/no’ basis, where ‘Yes’ indicated good practice and was scored ‘1’ and ‘No’ indicated bad practice and was scored ‘0’. The total practice score ranged from 0 to 10, with consideration of achieving more than 60% from total score as a good practice.

### Pilot study

Firstly, the content validity of the questionnaire was assessed by three Egyptian experts in the field of epidemiology and research to ascertain relevance and completeness. The experts’ responses to every question were represented in four points rating score ranging from 4 to 1, where 4 = strongly relevant, 3 = relevant, 2 = little relevant and 1 = not relevant. Then the summation of these responses was carried out and divided by the total score to calculate the percentage score. Validity of the questionnaire in view of experts’ conclusion was found to be 95%. Then the questionnaires were completed through telephone communication with contacts of COVID-19 cases traced in the first two areas where COVID-19 began to appear in Sharkia Governorate and excluded later from the study. The first area was chosen as a control group (number of contacts was 16) and the second as an experimental group (number of contacts 22). In the pilot study, we tried to simulate what would be done during the experiment to ensure applicability of the study. The Cronbach’s alpha coefficient of the questionnaire was 0.75, indicating an acceptable internal consistency.

### Fieldwork

Amongst the efforts in Egypt to control COVID-19 pandemic was the formulation of a collaborative protocol between the Ministry of Health and all Faculties of Medicine in the country. This protocol stated that once a confirmed COVID-19 case appears in any place, the faculty staff members will assist the LHAs in surveillance of this person’s contacts for 14 days from the last contact with this case (home isolation period). This surveillance depended only on an active daily monitoring for COVID-19 symptoms through phone calls. The control group was exposed to this routine surveillance.

The researchers as faculty staff members received the telephone numbers and the personal data of participants from the LHAs. They phoned them all but its frequency was different amongst the study groups. For the control group, phoning was done only twice at the beginning and one week after the home isolation period. For the experimental group, phoning was done all through the home isolation period and one week after it. All COVID-19 contacts appearing during the study period agreed to participate in the study, providing a response rate of 100%. The study passed through three phases, which are discussed next.

#### Pre-intervention phase

This phase was conducted on the second or third day of home isolation period where COVID-19 KAP questionnaire was completed through a telephone contact with each participant in both the experimental and control groups after taking consent.

#### Intervention phase

This was conducted during the home isolation period where the experimental group was exposed to telephone counselling via the researchers whose phone numbers were available to all participants to simulate the hot line. Telephone counselling composed of direct telephone conversations on scheduled alternative days and during any emergency and continuous WhatsApp contact through text messages or voice calls for any non-urgent question. During this counselling, the following was done: monitoring for COVID-19 symptoms; providing short tailored health education messages about the clinical presentations, transmission routes, prevention and control of COVID-19; providing psychological support and solving any health problems that appeared during the home isolation period and which might have led to the need for medical consultation and subsequently non-adherence to home isolation.

#### Post-intervention phase

This phase was conducted one week after the home isolation period where the same questionnaire of the pre-intervention phase was completed through telephone contact with each participant in both experimental and control groups.

### Statistical analysis

All the statistical analyses were carried out by using Statistical Package for Social Sciences (SPSS Inc., Illinois, United States) version 20.0. Chi-square, McNemar, Fisher’s exact tests and correlation co-efficient were measured. The statistical significance level was set at *p* < 0.05.

### Ethical considerations

The study was approved by Institutional Review Board (IRB) of the Faculty of Medicine, Zagazig University (reference no: ZU-IRB #6236-26-3-2020).

An official permission was obtained from the Health Directorate in Sharkia Governorate and informed verbal consent was obtained from all the participants after simple and clear explanation of the research objectives to them.

## Results

Regarding the socio-demographic characteristics of the participants, there were no statistically significant differences between experimental and control groups in all these variables (Table 2-A1).

Table 1 shows that after telephone counselling, the knowledge of the experimental group significantly improved compared with the control group in all questions covering COVID-19 epidemiology, infection prevention and control. The percent of COVID-19 contacts who achieved a good total knowledge score in the experimental group was 91.3% compared with 13.5% in the control group.

**TABLE 1:** COVID-19-related knowledge of the contacts of confirmed COVID-19 cases before and after intervention.

Knowledge items†	Pre-intervention				*χ*^2^	*p*	Post-intervention				*χ*^2^	*p*	McNemar	*p*
	Good knowledge		Bad knowledge				Good knowledge		Bad knowledge					
	No.	%	No.	%			No.	%	No.	%				
**K1**	-	-	-	-	2.18	0.21	-	-	-	-	100	0.00*	-	-
Experimental	12	66.7	6	33.3	-	-	85	86.7	19	17.3	-	-	0.09	0.00*
Control	92	48.4	98	51.6	-	-	13	13.3	91	82.7	-	-	-	-
**K2**	-	-	-	-	1.85	0.24	-	-	-	-	105	0.00*	-	-
Experimental	19	61.3	12	38.7	-	-	91	84.3	17	15.7	-	-	0.06	0.00*
Control	85	48.0	92	52.0	-	-	13	13.0	87	87.0	-	-	-	-
**K3**	-	-	-	-	2.4	0.18	-	-	-	-	68.7	0.00*	-	-
Experimental	15	65.2	8	34.8	-	-	73	83.9	31	25.6	-	-	0.07	0.00*
Control	89	48.1	96	51.8	-	-	14	16.1	90	74.4	-	-	-	-
**K4**	-	-	-	-	2.55	0.16	-	-	-	-	27.9	0.00*	-	-
Experimental	15	38.5	89	52.7	-	-	68	69.4	30	30.6	-	-	0.21	0.00*
Control	24	61.5	80	47.3	-	-	36	32.7	74	67.3	-	-	-	-
**K5**	-	-	-	-	2.2	0.2	-	-	-	-	74.9	0.00*	-	-
Experimental	16	64.0	9	36.0	-	-	77	83.7	15	16.3	-	-	0.03	0.00*
Control	88	48.1	95	51.9	-	-	27	23.3	89	76.7	-	-	-	-
**K6**	-	-	-	-	7.6	0.01*	-	-	-	-	111.2	0.00*	-	-
Experimental	19	76.0	6	24.0	-	-	88	88.0	12	12.0	-	-	0.18	0.00*
Control	85	46.4	98	53.6	-	-	16	14.8	92	85.2	-	-	-	-
**K7**	-	-	-	-	0.16	0.84	-	-	-	-	69.4	0.00*	-	
Experimental	16	53.3	14	46.7	-	-	79	80.6	19	19.4	-	-	0.30	0.00*
Control	88	49.4	90	50.6	-	-	25	22.7	85	77.3	-	-	-	-
**K8**	-	-	-	-	1.9	0.23	-	-	-	-	136	0.00*	-	-
Experimental	18	62.1	86	48.0	-	-	91	92.9	7	7.1	-	-	0.48	0.00*
Control	11	37.9	93	52.0	-	-	13	11.8	97	88.2	-	-	-	-
**K9**	-	-	-	-	2.4	0.18	-	-	-	-	89.9	0.00*	-	-
Experimental	15	65.2	8	34.8	-	-	75	90.4	8	9.6	-	-	1	0.00*
Control	89	48.1	96	51.9	-	-	29	23.4	96	76.8	-	-	-	-
**K10**	-	-	-	-	0.81	0.5	-	-	-	-	100.6	0.00*	-	-
Experimental	13	59.1	9	40.9	-	-	83	88.3	11	11.7	-	-	0.5	0.00*
Control	91	48.9	95	42.1	-	-	21	18.4	93	81.6	-	-	-	-
**K11**	-	-	-	-	2.1	0.25	-	-	-	-	93.9	0.00*	-	-
Experimental	9	69.2	4	30.8	-	-	74	92.5	6	7.5	-	-	0.62	0.00*
Control	95	48.7	100	51.3	-	-	30	23.4	98	76.6	-	-	-	-
**K12**	-	-	-	-	1.8	0.25	-	-	-	-	102.7	0.00*	-	-
Experimental	14	63.6	8	36.4	-	-	79	91.9	7	8.1	-	-	1	0.00*
Control	90	48.4	96	51.6	-	-	25	20.5	97	79.5	-	-	-	-
**Total knowledge**	-	-	-	-	0.47	0.64	-	-	-	-	126.4	**0.00**	-	-
Experimental	12	11.5	9	8.6	-	-	95	91.3	14	13.5	-	-	0.35	0.00*
Control	92	88.5	95	91.4	-	-	9	8.7	90	86.5	-	-	-	-

Note: Information in bold is statistically significant.

* , Statistically significant *p*-value < 0.05.

† , Knowledge items are illustrated in Table 1-A1.

Table 2 shows that after telephone counselling, all attitudes of the experimental group significantly improved compared with the control group. A total of 57.8% COVID-19 contacts achieved positive total attitude score in the experimental group compared with 7.8% in the control group.

**TABLE 2:** COVID-19-related attitude of the contacts of confirmed COVID-19-cases before and after intervention.

Attitude items†	Pre-intervention				*χ*^2^	*p*	Post-intervention				*χ*^2^	*p*	McNemar	*p*
	Negative attitude		Positive attitude				Negative attitude		Positive attitude					
	No.	%	No.	%			No.	%	No.	%				
**A1**	-	-	-	-	3.7	0.07	-	-	-	-	4.7	0.04*	-	-
Experimental	45	43.3	59	56.7	-	-	22	37.9	36	62.1	-	-	0.19	0.00*
Control	59	56.7	45	43.3	-	-	82	54.7	68	45.3	-	-	-	
**A2**	-	-	-	-	2.9	0.11	-	-	-	-	9.3	0.00*	-	-
Experimental	71	54.6	33	42.3	-	-	42	39.6	64	60.4	-	-	0.5	0.00*
Control	59	45.4	45	57.7	-	-	62	60.8	40	39.2	-	-	-	-
**A3**	-	-	-	-	0.59	0.56	-	-	-	-	69.6	0.00*	-	-
Experimental	90	51.1	86	48.9	-	-	18	18.8	78	81.2	-	-	0.07	0.00*
Control	14	43.8	18	56.2	-	-	86	76.8	26	23.2	-	-	-	-
**A4**	-	-	-	-	0.24	0.74	-	-	-	-	44.7	0.00*	-	-
Experimental	78	49.1	26	53.1	-	-	30	28.9	81	71.1	-	-	1	0.00*
Control	81	50.9	23	46.9	-	-	74	75.5	23	24.5	-	-	-	-
**Total attitude**	-	-	-	-	0.41	0.67	-	-	-	-	58.2	0.00*	-	-
Experimental	93	89.4	11	10.6	-	-	43	41.3	95	91.3	-	-	0.3	0.00*
Control	90	86.5	14	13.5	-	-	61	58.7	9	8.7	-	-	-	-

Note: Information in bold is statistically significant.

* , Statistically significant *p*-value < 0.05.

† , attitudes items were illustrated in Table 1-A1.

Table 3 shows that after telephone counselling, all health practices of the experimental group significantly improved compared with the control group. A total of 71.2% COVID-19 contacts achieved a good total practice score in the experimental group compared with 16.3% in the control group.

**TABLE 3:** Health practices of the contacts of confirmed COVID-19 cases before and after intervention.

Practice items†	Pre-intervention				*χ*^2^	*p*	Post-intervention				*χ*^2^	*p*	McNemar	*p*
	Good practice		Bad practice				Good practice		Bad practice					
	No.	%	No.	%			No.	%	No.	%				
**P1**	-	-	-	-	0.59	0.56	-	-	-	-	40.3	0.00*	-	-
Experimental	18	56.2	86	48.9	-	-	65	76.5	39	31.7	-	-	0.07	0.00*
Control	14	43.8	90	51.1	-	-	20	23.5	84	68.3	-	-	-	-
**P2**	-	-	-	-	0.036	1.00	-	-	-	-	83.9	0.00*	-	-
Experimental	17	51.1	87	49.7	-	-	87	80.6	17	17.0	-	-	0.33	0.00*
Control	16	48.5	88	50.3	-	-	21	19.4	83	83.0	-	-	-	-
**P3**	-	-	-	-	0.165	0.83	-	-	-	-	78.8	0.00*	-	-
Experimental	15	53.6	89	49.4	-	-	86	79.6	18	18.0	-	-	0.07	0.00*
Control	13	46.4	91	50.6	-	-	22	20.4	82	82.0	-	-	-	-
P4	-	-	-	-	0.033	1.00	-	-	-	-	89.2	0.00*	-	-
Experimental	19	51.4	85	49.7	-	-	89	80.9	15	15.3	-	-	0.63	0.00*
Control	18	48.6	86	50.3	-	-	21	19.1	83	84.7	-	-	-	-
**P5**	-	-	-	-	1.4	0.32	-	-	-	-	66.9	0.00*	-	-
Experimental	12	40.0	92	51.7	-	-	82	78.1	22	21.4	-	-	0.23	0.00*
Control	18	60.0	86	48.3	-	-	23	21.9	81	78.6	-	-	-	-
**P6**	-	-	-	-	1.1	0.37	-	-	-	-	58.3	0.00*	-	-
Experimental	17	42.5	87	51.8	-	-	82	75.2	22	22.2	-	-	0.29	0.00*
Control	23	57.5	81	48.2	-	-	27	24.8	77	77.8	-	-	-	-
**P7**	-	-	-	-	0.51	0.59	-	-	-	-	84.0	0.00*	-	-
Experimental	21	55.3	83	48.8	-	-	88	80.0	16	16.3	-	-	0.12	0.00*
Control	17	44.7	87	51.2	-	-	22	20.0	82	83.7	-	-	-	-
**P8**	-	-	-	-	4.6	0.05	-	-	-	-	63.5	0.00*	-	-
Experimental	25	65.8	79	46.5	-	-	74	81.3	30	25.6	-	-	0.42	0.00*
Control	13	34.2	91	53.5	-	-	17	18.7	87	74.4	-	-	-	-
**P9**	-	-	-	-	0.7	0.50	-	-	-	-	72.1	0.00*	-	-
Experimental	15	57.7	89	48.9	-	-	78	82.1	26	23.0	-	-	0.10	0.00*
Control	11	42.3	93	51.1	-	-	17	17.9	87	77.0	-	-	-	-
**P10**	-	-	-	-	3.8	0.07	-	-	-	-	88.9	0.00*	-	-
Experimental	20	66.7	84	47.2	-	-	85	83.3	19	17.9	-	-	0.11	0.00*
Control	10	33.3	94	52.8	-	-	17	16.7	87	82.1	-	-	-	-
**P11**	-	-	-	-	3.6	0.09	-	-	-	-	44.9	0.00*	-	-
Experimental	23	63.9	81	47.1	-	-	70	76.1	34	29.3	-	-	0.12	0.00*
Control	13	36.1	91	52.9	-	-	22	23.9	82	70.7	-	-	-	-
**Total practice**	-	-	-	-	3.4	0.07	-	-	-	-	63.4	0.00*	-	-
Experimental	20	19.2	10	9.6	-	-	74	71.2	30	28.8	-	-	0.19	0.00*
Control	84	80.8	94	90.4	-	-	17	16.3	87	83.7	-	-	-	-

Note: Information in bold is statistically significant.

* , Statistically significant *p*-value < 0.05.

† , Practice items are illustrated in Table 1-A1.

Tables 1–3 also show insignificant differences in knowledge, attitudes and health practices of the experimental and control groups at baseline. They also show insignificant changes in knowledge, attitudes and health practices of the control group despite of surveillance with LHA.

Table 4 demonstrates a statistically insignificant association between all socio-demographic factors of the experimental group and the post-intervention KAP scores except the male gender and working group that were associated with bad practice score.

**TABLE 4:** Relationship between socio-demographic characters of the experimental group and post-intervention knowledge, attitude and practice.

Socio-demographic characters	Knowledge				*χ*^2^	*P*	Attitude				*χ*^2^	*P*	Practice				*χ*^2^	*P*
	Good†		Bad‡				Positive§		Negative¶				Good††		Bad‡‡			
	*n*	%	*n*	%			*n*	%	*n*	%			*n*	%	*n*	%		
**Age (year)**	-	-	-	-	-	0.26§§	-	-	-	-	2.2	0.13	-	-	-	-	1.7	0.18
18–˂ 45	61	64.2	34	35.8	-	-	44	72.1	17	27.9	-	-	52	70.3	22	29.7	-	-
45–69	8	88.9	1	11.1	-	-	25	58.1	18	41.9	-	-	17	56.7	13	43.3	-	-
**Gender**	-	-	-	-	-	0.72§§	-	-	-	-	0.05	0.80	-	-	-	-	4.7	0.02*
Male	53	55.8	42	44.2	-	-	34	72.1	27	27.9	-	-	37	50.0	37	50.0	-	-
Female	6	66.7	3	33.3	-	-	25	62.8	18	37.2	-	-	22	73.3	8	26.7	-	-
**Residence**	-	-	-	-	-	1.00§§	-	-	-	-	0.06	0.80	-	-	-	-	3.5	0.05
Rural	74	77.9	21	22.1	-	-	47	77.0	14	23.0	-	-	54	73.0	20	27.0	-	-
Urban	7	77.8	2	22.2	-	-	34	79.1	9	20.9	-	-	27	90.0	3	10.0	-	-
**Marital status**	-	-	-	-	-	0.15§§	-	-	-	-	0.92	0.33	-	-	-	-	0.8	0.35
Unmarried	36	37.9	59	62.1	-	-	27	44.3	34	55.7	-	-	32	43.2	42	56.8	-	-
Married	6	66.7	3	33.3	-	-	15	34.9	28	65.1	-	-	10	33.3	20	66.7	-	-
**Education**	-	-	-	-	-	0.17§§	-	-	-	-	-	0.05§§	-	-	-	-	-	0.19§§
Illiterate/low	23	23.2	4	33.3	-	-	17	18.0	32	60.5	-	-	18	20.3	20	56.7	-	-
Middle	5	66.7	7	7.4	-	-	11	39.5	7	11.5	-	-	10	43.3	7	9.5	-	-
High	65	69.5	0	0.00	-	-	37	70.5	0	0.0	-	-	49	70.3	0	0.0	-	-
**Occupation**	-	-	-	-	-	0.71§§	-	-	-	-	1.01	0.31	-	-	-	-	4.4	0.03*
Yes	64	67.4	31	32.6	-	-	44	72.1	17	27.9	-	-	46	62.2	28	37.8	-	-
No	7	77.8	2	22.2	-	-	27	62.8	16	37.2	-	-	25	83.3	5	16.7	-	-
**Social class**	-	-	-	-	-	0.14§§	-	-	-	-	3.5	0.16	-	-	-	-	3.4	0.17
Low	37	36.8	3	11.1	-	-	27	27.9	22	27.9	-	-	32	43.2	19	63.3	-	-
Medium	3	55.6	10	10.5	-	-	13	53.5	5	8.2	-	-	8	26.7	10	13.6	-	-
High	48	52.6	3	33.3	-	-	29	63.9	8	18.6	-	-	32	43.2	3	10.0	-	-

* , Statistically significant *p*-value < 0.05.

† , *n* = 95;

‡ , *n* = 9;

§ , *n* = 61;

¶ , *n* = 43;

†† , *n* = 74;

‡‡ , *n* = 30;

§§ , Fisher’s exact test was used.

Table 5 reveals a statistically significant positive correlation between differences in knowledge, attitudes and practices of the experimental group before and after telephone counselling.

**TABLE 5:** Correlation between differences in knowledge, attitude and practice of the experimental group before and after intervention.

Items	Knowledge difference	Attitude difference	Practice difference
Knowledge difference			
*r*	1.000	0.522*	0.813*
*p*	-	**0.000**	**0.000**
Attitude difference			
*r*	0.522*	1.000	0.458*
*p*	**0.000**	-	**0.000**
Practice difference			
*r*	0.813*	0.458*	1.000
*p*	**0.000**	**0.000**	-

Note: Information in bold is statistically significant.

* , Correlation is significant at the 0.01 level (2-tailed).

## Discussion

Our study showed that telephone counselling was effective in improving the experimental group’s knowledge about COVID-19, decreasing their worry about getting COVID-19 and being exposed to its related stigma and helping them to adhere to the recommended preventative practices. This result is consistent with the study conducted by Chan et al. in China during the severe acute respiratory syndrome (SARS) pandemic, which found that telephone health education seemed to be effective in producing significant changes of knowledge, relieving anxiety related to the SARS pandemic and improving the practice of identified preventative measures related to SARS.^14^ From this research it would appear that the lessons learned from the SARS pandemic about the value of telephone counselling can be applied during the COVID-19 pandemic. Furthermore, the WHO reported that increase in public awareness about the emerging disease played an important role in relieving negative social phenomena such as anxiety, fear and stigmatisation.^15^ Telephone counselling can be one of the used methods in increasing public awareness during a pandemic.

The effectiveness of telephone counselling can be shown through the following study results: firstly, the statistically insignificant differences between experimental and control groups in all socio-demographic characteristics, which confirms that there was no selection bias; secondly, the statistically insignificant differences between experimental and control groups in baseline KAP scores related to COVID-19, which became statistically significant after telephone counselling; finally, the presence of insignificant changes in KAP scores of the control group in spite of surveillance with LHAs. Local health authorities depended only on an active daily monitoring for COVID-19 symptoms during surveillance of COVID-19 contacts and neglected the practice of health education. This might be attributed to their implicit dependence on health education provided by the Ministry of Health through mass media and official websites without assuring whether this health education is accessible to everyone or satisfies the needs of everyone.

Before telephone counselling, our study revealed low level of knowledge, negative attitude and bad practice towards COVID-19 in both the experimental and control groups. This could be attributed to two factors: firstly, our study was conducted early at the beginning of appearance of COVID-19 at Sharkia Governorate, with low expectation and preparedness for the disease. This is consistent with other studies that reported that the general public lacked good KAP of SARS and Middle East respiratory syndrome (MERS) at the early stage of epidemics.^16,17^ Secondly, the majority of our participants in both groups were either illiterate or below university education, which will surely affect their KAP. This result is contrary to other studies conducted in China, Egypt and Iran, which used online surveys to assess the KAP of general public towards COVID-19 and which reported high level of knowledge, appropriate practice and positive attitude towards COVID-19.^12,18,19^ This difference in results may be because of dependence of these studies on online surveys, which are usually accessible for participants with university education. This reflects that although the use of online surveys is an important precautionary measure during the epidemics, the data revealed from them cannot be generalised to the whole population and they should be replaced by more widespread methods, for example, telephone survey.

Our study also revealed that telephone counselling was effective in improving the KAP scores of all participants in the experimental group regardless of their socio-demographic factors except the male gender and working group, which were associated with bad practice score. This could be explained by the fact that men are usually representative of the working group and working conditions may hinder the adherence to home isolation and other preventive practices. Furthermore, our study revealed a statistically significant positive correlation between differences in knowledge, attitudes and practices of the experimental group before and after the intervention and this assures us that effective changes can be produced by telephone counselling.

Limitations of this study included a lack of long-term follow-up that may be needed to track the maintenance of preventive behaviour. Therefore, we recommend that further studies should be conducted for longer periods of time to prove the long-term effect of telephone counselling.

## Conclusion and recommendation

Our study demonstrated the effectiveness of telephone counselling, which incorporated active monitoring for COVID-19 symptoms, health education, psychological support and problem-solving in improving the knowledge, attitudes and practices of COVID-19 contacts. Conversely, routine surveillance by LHAs that depended only on an active daily monitoring for COVID-19 symptoms through phone contact was not sufficient to change the knowledge, attitudes and practices of COVID-19 contacts. This suggests that health authorities should be more aware of the potential of telephone counselling during surveillance of COVID-19 contacts as an accessible, safe and reliable method to improve their KAP about this emerging disease.

## Appendix 1

**TABLE 1-A1:** Parts II, III and IV of COVID-19 knowledge, attitude and practice questionnaire.

Questions	Options
**Part II: Knowledge**	
K1. The main clinical symptoms of COVID-19 are increased body temperature, fatigue, dry cough and myalgia.	True, false, I don’t know
K2. Unlike the common cold, stuffy nose, runny nose and sneezing are less popular in persons infected with the COVID-19 virus.	True, false, I don’t know
K3. There is currently no effective treatment for COVID-19, but early symptomatic and supportive treatment can aid most patients recover from the infection.	True, false, I don’t know
K4. Not all persons with COVID-19 will develop to severe cases. Only those who are elderly have chronic illnesses and are obese are more likely to be severe cases.	True, false, I don’t know
K5. Eating or contacting wild animals would result in the infection by the COVID-19 virus.	True, false, I don’t know
K6. Persons with COVID-19 cannot transmit the infection with the virus to others when a fever is not present.	True, false, I don’t know
K7. The COVID-19 virus spreads via respiratory droplets of infected individuals.	True, false, I don’t know
K8. Ordinary residents can wear general medical masks to prevent the infection by the COVID-19 virus.	True, false, I don’t know
K9. It is not necessary for children and young adults to take measures to prevent the infection by the COVID-19 virus.	True, false, I don’t know
K10. To prevent the infection by COVID-19, individuals should avoid going to crowded places such as train stations and avoid taking public transportations.	True, false, I don’t know
K11. Isolation and treatment of people who are infected with the COVID-19 virus are effective ways to reduce the spread of the virus.	True, false, I don’t know
K12. People who have contact with someone infected with the COVID-19 virus should be immediately isolated in a proper place. In general, the observation period is 14 days.	True, false, I don’t know
**Part III: Attitudes**	
A1. Do you worry about getting COVID-19 infection?	Yes, no
A2. Do you worry about people talking badly about you because you are a contact of COVID-19 case?	Yes, no
A3. Do you worry about being avoided by friends and family because you are a contact of COVID-19 case?	Yes, no
A4. Do you worry about seeking medical care if you have a fever, cough and difficulty breathing?	Yes, no
**Part VI: Practices**	
P1. Have you adhered to voluntary home quarantine (self-isolation) during the 14 days from last contact with confirmed COVID-19 case?	Yes, no
P2. Have you monitored yourself for COVID-19 symptoms?	Yes, no
P3. Will you seek medical care early if you have a fever, cough and difficulty breathing?	Yes, no
P4. Have you washed hands frequently with water and soap or used hand-sanitising gel?	Yes, no
P5. Have you followed respiratory etiquette?	Yes, no
P6. Have you avoided touching eyes, nose and mouth?	Yes, no
P7. Have you regularly cleaned surfaces and flour at home by sanitisers?	Yes, no
P8. Have you gone to any crowded place?	Yes, no
P9. If you went to any crowded place, you would maintain social distancing (keeping a distance of 1 m between yourself and anyone who is coughing or sneezing)?	Yes, no
P10. Have you stayed informed and followed the advice given by your healthcare provider, national and local public health authority, or your employer on how to protect yourself and others from COVID-19?	Yes, no
P11. Have you used a mask when leaving home?	Yes, no

*Source:* Adapted from Zhong B, Luo W, Li H, et al. Knowledge, attitudes, and practices towards COVID-19 among Chinese residents during the rapid rise period of the COVID-19 outbreak: A quick online cross-sectional survey. Int J Biol Sci. 2020;16(10):1745–1752. https://doi.org/10.7150/ijbs.45221

COVID-19, coronavirus disease 2019.

**TABLE 2-A1:** Socio-demographic characteristics of experimental and control groups.

Items	Experimental group†		Control group‡		*χ*^2^	*p*
	*n*	%	*n*	%		
**Age (year)**						
18–˂45	69	66.4	57	54.8	2.8	0.08
45–69	35	33.6	47	45.2		
**Gender**						
Male	59	56.7	49	47.1	1.92	0.16
Female	45	43.3	55	52.9		
**Residence**						
Rural	81	77.9	69	66.3	3.4	0.06
Urban	23	22.1	35	33.7		
**Marital status**						
Unmarried	42	40.4	55	52.9	3.2	0.07
Married	62	59.6	49	47.1		
**Education**						
Illiterate and low	28	26.9	36	34.6	1.6	0.44
Middle	69	66.4	63	60.6		
High	7	6.7	5	4.8		
**Occupation**						
Yes	71	68.3	59	56.7	2.9	0.08
No	33	31.7	45	43.3		
**Social class**						
Low	40	38.5	49	47.1	1.6	0.43
Medium	51	49.0	45	43.3		
High	13	12.5	10	9.6		

† , *n* = 104;

‡ , *n* = 104.
